# *N*-(3-Amino­bicyclo­[2.2.1]heptan-2-yl)-4-methyl­benzene­sulfonamide

**DOI:** 10.1107/S1600536812025421

**Published:** 2012-06-13

**Authors:** Alaa A.-M. Abdel-Aziz, Adel S. El-Azab, Magda A. El-Sherbeny, Seik Weng Ng, Edward R. T. Tiekink

**Affiliations:** a Department of Pharmaceutical Chemistry, College of Pharmacy, King Saud University, Riyadh 11451, Saudi Arabia; b Department of Medicinal Chemistry, Faculty of Pharmacy, University of Mansoura, Mansoura 35516, Egypt; c Department of Organic Chemistry, Faculty of Pharmacy, Al-Azhar University, Cairo 11884, Egypt; d Department of Chemistry, University of Malaya, 50603 Kuala Lumpur, Malaysia; e Chemistry Department, Faculty of Science, King Abdulaziz University, PO Box 80203 Jeddah, Saudi Arabia

## Abstract

In the title compound, C_14_H_20_N_2_O_2_S, the sulfonamide O atoms lie to one side of the benzene ring and the amino­bicyclo­hepta­nyl to the other side [C_ar_—S—N—C torsion angle = −57.93 (11)°; ar = aromatic]. An intra­molecular N—H⋯N hydrogen bond is formed. In the crystal, a supra­molecular chain is formed along the *b* axis *via* N—H⋯O and N—H⋯N hydrogen bonds.

## Related literature

For chiral ligands in asymmetric catalytic reactions, see: Seo *et al.* (2001 ▶); Abdel-Aziz *et al.* (2004 ▶); Matsunaga *et al.* (2005 ▶); Yamakuchi *et al.* (2005 ▶).
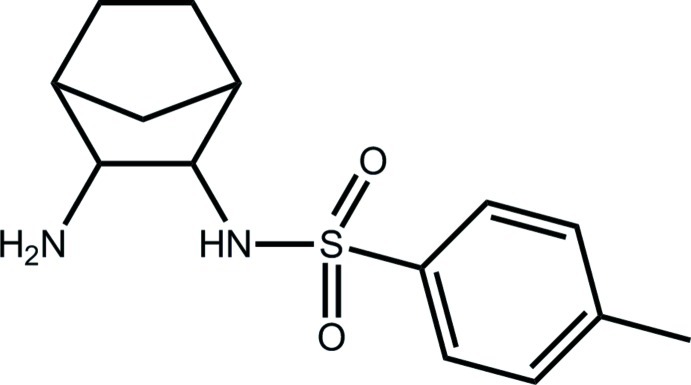


## Experimental

### 

#### Crystal data

C_14_H_20_N_2_O_2_S
*M* *_r_* = 280.38
Monoclinic, 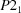

*a* = 10.1715 (2) Å
*b* = 6.1169 (1) Å
*c* = 11.5150 (3) Å
β = 110.332 (2)°
*V* = 671.80 (2) Å^3^
*Z* = 2
Cu *K*α radiation
μ = 2.14 mm^−1^
*T* = 100 K
0.30 × 0.20 × 0.10 mm

#### Data collection

Agilent SuperNova Dual diffractometer with Atlas detector
Absorption correction: multi-scan (*CrysAlis PRO*; Agilent, 2012 ▶) *T*_min_ = 0.566, *T*_max_ = 0.814
4721 measured reflections
2750 independent reflections
2731 reflections with *I* > 2σ(*I*)
*R*_int_ = 0.014

#### Refinement

*R*[*F* ^2^ > 2σ(*F* ^2^)] = 0.024
*wR*(*F* ^2^) = 0.066
*S* = 1.03
2750 reflections
185 parameters
4 restraints
H atoms treated by a mixture of independent and constrained refinement
Δρ_max_ = 0.22 e Å^−3^
Δρ_min_ = −0.26 e Å^−3^
Absolute structure: Flack (1983 ▶), 1217 Friedel pairs
Flack parameter: −0.001 (10)

### 

Data collection: *CrysAlis PRO* (Agilent, 2012 ▶); cell refinement: *CrysAlis PRO*; data reduction: *CrysAlis PRO*; program(s) used to solve structure: *SHELXS97* (Sheldrick, 2008 ▶); program(s) used to refine structure: *SHELXL97* (Sheldrick, 2008 ▶); molecular graphics: *ORTEP-3* (Farrugia, 1997 ▶) and *DIAMOND* (Brandenburg, 2006 ▶); software used to prepare material for publication: *publCIF* (Westrip, 2010 ▶).

## Supplementary Material

Crystal structure: contains datablock(s) global, I. DOI: 10.1107/S1600536812025421/lh5485sup1.cif

Structure factors: contains datablock(s) I. DOI: 10.1107/S1600536812025421/lh5485Isup2.hkl

Supplementary material file. DOI: 10.1107/S1600536812025421/lh5485Isup3.cml

Additional supplementary materials:  crystallographic information; 3D view; checkCIF report

## Figures and Tables

**Table 1:** Hydrogen-bond geometry (Å, °)

*D*—H⋯*A*	*D*—H	H⋯*A*	*D*⋯*A*	*D*—H⋯*A*
N1—H1*n*⋯O1^i^	0.88 (1)	2.20 (1)	2.976 (2)	148 (2)
N1—H2*n*⋯N2	0.87 (1)	2.39 (2)	2.752 (2)	105 (2)
N2—H3*n*⋯N1^i^	0.89 (1)	2.04 (1)	2.907 (2)	166 (2)

Symmetry code: (i) 
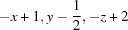
.

**Fig. 1.**
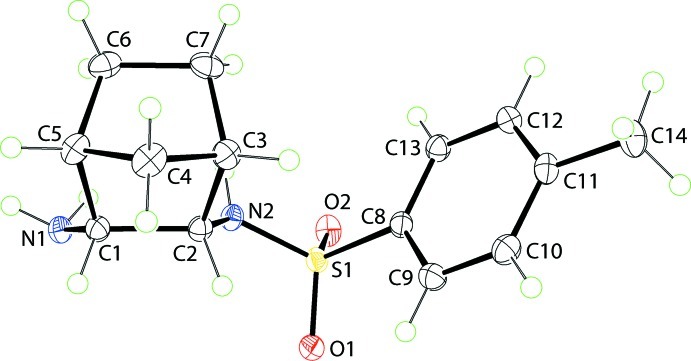
The molecular structure of (I) showing the atom-labelling scheme and displacement ellipsoids at the 50% probability level.

**Fig. 2.**
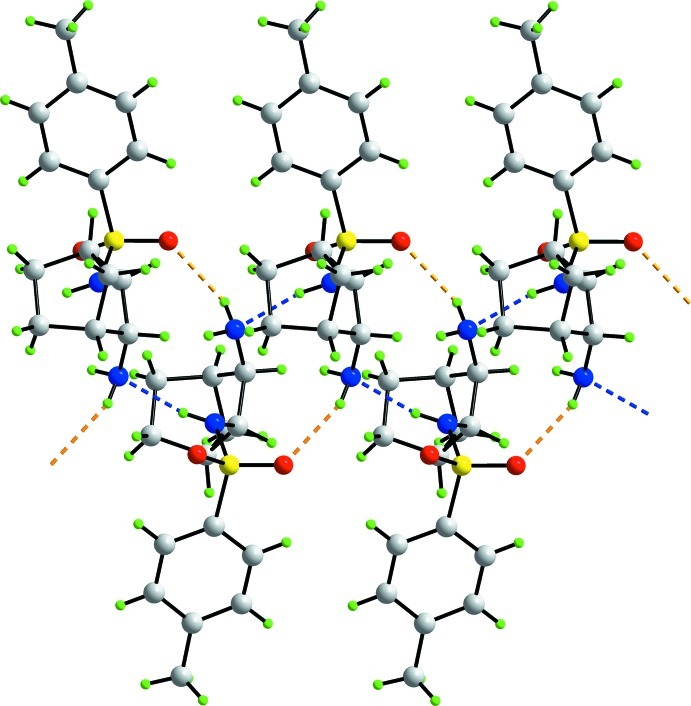
A view of the supramolecular helical chain along the b axis in (I). The N—H···O and N—H···N hydrogen bonds are shown as orange and blue dashed lines, respectively.

**Fig. 3.**
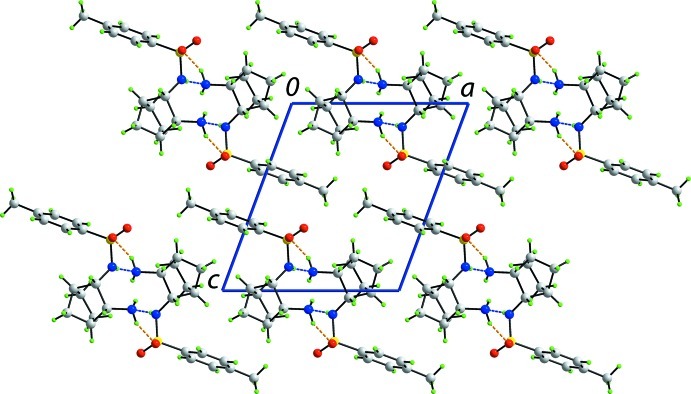
A view in projection down the b axis of the unit-cell contents for (I). The N—H···O and N—H···N (obscured) hydrogen bonds are shown as orange and blue dashed lines, respectively.

## supplementary crystallographic information

### Comment

The title compound (I) was synthesized in the context of the development of chiral ligands for asymmetric catalytic reactions (Seo *et al.*, 2001; Abdel-Aziz *et al.*, 2004; Matsunaga *et al.*, 2005; Yamakuchi *et al.* 2005).

In (I), Fig. 1, the two S-bound O atoms lie to one side of the adjacent benzene ring with the O1 and O2 atoms lying -0.466 (1) and -0.771 (1) Å out of the plane, respectively, and the aminobicycloheptanyl residue lying to the other side, the C8—S1—N2—C2 torsion being -57.93 (11)°. An intramolecular N—H···N hydrogen bond is noted, Table 1.

Molecules are connected into a supramolecular chain along the *b* axis *via* N—H···O and N—H···N hydrogen bonds that generate 12-membered {···HNC_2_NH···OSNC_2_N} synthons, Fig. 2 and Table 1. The chains pack into a three-dimensional architecture without specific interactions between them, Fig. 3.

### Experimental

To the mixture of 2-imidazolidinone (2.0 ml), water (2 ml), ethanol (6 ml) and Ba(OH)_2_.8H_2_O (20 ml) were added. This was heated at 413 K in a glass sealed tube for 24 h. The solvents were evaporated and the precipitate extracted three times with chloroform (10 ml × 3). The organic extract was dried and crystallized from ethanol to afford the title compound.

### Refinement

Carbon-bound H-atoms were placed in calculated positions [C—H = 0.95 to 1.00 Å, *U*_iso_(H) = 1.2–1.5*U*_eq_(C)] and were included in the refinement in the riding model approximation. The amino H-atoms were refined with N—H = 0.88±0.01 Å.

### Crystal data

**Table:**

C_14_H_20_N_2_O_2_S	*F*(000) = 300
*M**_r_* = 280.38	*D*_x_ = 1.386 Mg m^−^^3^
Monoclinic, *P*2_1_	Cu *K*α radiation, λ = 1.54184 Å
Hall symbol: P 2yb	Cell parameters from 3574 reflections
*a* = 10.1715 (2) Å	θ = 4.1–76.4°
*b* = 6.1169 (1) Å	µ = 2.14 mm^−^^1^
*c* = 11.5150 (3) Å	*T* = 100 K
β = 110.332 (2)°	Prism, colourless
*V* = 671.80 (2) Å^3^	0.30 × 0.20 × 0.10 mm
*Z* = 2	

### Data collection

**Table:**

Agilent SuperNova Dual diffractometer with Atlas detector	2750 independent reflections
Radiation source: SuperNova (Cu) X-ray Source	2731 reflections with *I* > 2σ(*I*)
Mirror monochromator	*R*_int_ = 0.014
Detector resolution: 10.4041 pixels mm^-1^	θ_max_ = 76.6°, θ_min_ = 4.1°
ω scan	*h* = −11→12
Absorption correction: multi-scan (*CrysAlis PRO*; Agilent, 2012)	*k* = −7→7
*T*_min_ = 0.566, *T*_max_ = 0.814	*l* = −14→12
4721 measured reflections	

### Refinement

**Table:**

Refinement on *F*^2^	Secondary atom site location: difference Fourier map
Least-squares matrix: full	Hydrogen site location: inferred from neighbouring sites
*R*[*F*^2^ > 2σ(*F*^2^)] = 0.024	H atoms treated by a mixture of independent and constrained refinement
*wR*(*F*^2^) = 0.066	*w* = 1/[σ^2^(*F*_o_^2^) + (0.049*P*)^2^ + 0.0858*P*] where *P* = (*F*_o_^2^ + 2*F*_c_^2^)/3
*S* = 1.03	(Δ/σ)_max_ = 0.001
2750 reflections	Δρ_max_ = 0.22 e Å^−^^3^
185 parameters	Δρ_min_ = −0.26 e Å^−^^3^
4 restraints	Absolute structure: Flack (1983), 1217 Friedel pairs
Primary atom site location: structure-invariant direct methods	Flack parameter: −0.001 (10)

### Fractional atomic coordinates and isotropic or equivalent isotropic displacement parameters (Å^2^)

**Table:**

	*x*	*y*	*z*	*U*_iso_*/*U*_eq_	
S1	0.26041 (3)	0.50378 (5)	0.72818 (2)	0.01602 (9)	
O1	0.26695 (10)	0.73734 (17)	0.71708 (9)	0.0218 (2)	
N1	0.52705 (11)	0.5254 (2)	1.10027 (10)	0.0184 (2)	
N2	0.32587 (11)	0.44016 (18)	0.87334 (10)	0.0160 (2)	
O2	0.32988 (10)	0.36603 (18)	0.66642 (9)	0.0215 (2)	
C1	0.38324 (13)	0.5685 (2)	1.09495 (11)	0.0168 (3)	
H1	0.3790	0.7246	1.1192	0.020*	
C2	0.26899 (13)	0.5369 (2)	0.96230 (11)	0.0153 (2)	
H2	0.2286	0.6832	0.9304	0.018*	
C3	0.15457 (14)	0.4000 (2)	0.98927 (13)	0.0190 (3)	
H3	0.0587	0.4149	0.9256	0.023*	
C4	0.16743 (13)	0.4842 (3)	1.11857 (12)	0.0224 (3)	
H4A	0.1085	0.4015	1.1559	0.027*	
H4B	0.1486	0.6429	1.1196	0.027*	
C5	0.32467 (14)	0.4279 (2)	1.17705 (12)	0.0198 (3)	
H5	0.3666	0.4604	1.2676	0.024*	
C6	0.32835 (14)	0.1852 (2)	1.14384 (13)	0.0213 (3)	
H6A	0.3138	0.0901	1.2079	0.026*	
H6B	0.4187	0.1470	1.1345	0.026*	
C7	0.20485 (14)	0.1631 (2)	1.01838 (13)	0.0220 (3)	
H7A	0.2373	0.1024	0.9533	0.026*	
H7B	0.1296	0.0687	1.0268	0.026*	
C8	0.07959 (14)	0.4382 (2)	0.67418 (11)	0.0164 (2)	
C9	−0.01687 (14)	0.6042 (2)	0.66650 (12)	0.0200 (3)	
H9	0.0144	0.7488	0.6912	0.024*	
C10	−0.15935 (14)	0.5562 (2)	0.62230 (12)	0.0205 (3)	
H10	−0.2253	0.6691	0.6170	0.025*	
C11	−0.20671 (13)	0.3446 (2)	0.58568 (12)	0.0185 (3)	
C12	−0.10771 (14)	0.1807 (2)	0.59531 (12)	0.0190 (3)	
H12	−0.1387	0.0356	0.5715	0.023*	
C13	0.03540 (14)	0.2258 (2)	0.63911 (12)	0.0180 (3)	
H13	0.1017	0.1131	0.6449	0.022*	
C14	−0.36173 (14)	0.2973 (3)	0.53354 (14)	0.0244 (3)	
H14A	−0.4059	0.3911	0.4615	0.037*	
H14B	−0.4036	0.3264	0.5969	0.037*	
H14C	−0.3765	0.1436	0.5083	0.037*	
H1n	0.5811 (16)	0.480 (3)	1.1733 (11)	0.025 (4)*	
H2n	0.530 (2)	0.412 (2)	1.0552 (16)	0.033 (5)*	
H3n	0.357 (2)	0.3032 (19)	0.882 (2)	0.035 (5)*	

### Atomic displacement parameters (Å^2^)

**Table:**

	*U*^11^	*U*^22^	*U*^33^	*U*^12^	*U*^13^	*U*^23^
S1	0.01351 (14)	0.01864 (15)	0.01503 (14)	−0.00197 (11)	0.00386 (10)	−0.00003 (11)
O1	0.0213 (5)	0.0204 (5)	0.0199 (5)	−0.0048 (4)	0.0026 (4)	0.0027 (4)
N1	0.0138 (5)	0.0199 (5)	0.0187 (5)	−0.0012 (5)	0.0021 (4)	0.0008 (5)
N2	0.0140 (5)	0.0182 (5)	0.0151 (5)	0.0023 (4)	0.0040 (4)	0.0002 (4)
O2	0.0177 (5)	0.0296 (5)	0.0185 (4)	0.0000 (4)	0.0081 (4)	−0.0028 (4)
C1	0.0169 (6)	0.0155 (6)	0.0161 (6)	0.0013 (4)	0.0032 (4)	−0.0005 (4)
C2	0.0141 (5)	0.0153 (6)	0.0160 (5)	0.0026 (4)	0.0046 (4)	0.0002 (5)
C3	0.0133 (6)	0.0223 (6)	0.0217 (6)	0.0008 (5)	0.0064 (5)	0.0013 (5)
C4	0.0201 (6)	0.0266 (7)	0.0240 (6)	0.0055 (6)	0.0122 (5)	0.0031 (6)
C5	0.0196 (6)	0.0226 (6)	0.0176 (6)	0.0041 (5)	0.0069 (5)	0.0024 (5)
C6	0.0198 (6)	0.0204 (7)	0.0259 (7)	0.0021 (5)	0.0106 (5)	0.0057 (5)
C7	0.0180 (6)	0.0197 (6)	0.0306 (7)	−0.0037 (5)	0.0113 (5)	0.0006 (6)
C8	0.0151 (6)	0.0188 (6)	0.0141 (6)	−0.0009 (5)	0.0036 (4)	0.0007 (4)
C9	0.0195 (6)	0.0174 (6)	0.0216 (6)	−0.0002 (5)	0.0053 (5)	−0.0012 (5)
C10	0.0178 (6)	0.0217 (7)	0.0216 (6)	0.0033 (5)	0.0063 (5)	0.0020 (5)
C11	0.0155 (6)	0.0247 (7)	0.0146 (6)	−0.0006 (5)	0.0041 (5)	0.0001 (5)
C12	0.0189 (6)	0.0183 (6)	0.0177 (6)	−0.0022 (5)	0.0037 (5)	−0.0010 (5)
C13	0.0171 (6)	0.0182 (6)	0.0175 (6)	0.0023 (5)	0.0046 (5)	−0.0003 (5)
C14	0.0154 (6)	0.0326 (8)	0.0238 (7)	−0.0015 (5)	0.0049 (5)	−0.0024 (6)

### Geometric parameters (Å, º)

**Table:**

S1—O2	1.4367 (10)	C5—H5	1.0000
S1—O1	1.4380 (11)	C6—C7	1.556 (2)
S1—N2	1.6172 (11)	C6—H6A	0.9900
S1—C8	1.7707 (13)	C6—H6B	0.9900
N1—C1	1.4665 (16)	C7—H7A	0.9900
N1—H1n	0.875 (9)	C7—H7B	0.9900
N1—H2n	0.873 (9)	C8—C13	1.3884 (18)
N2—C2	1.4649 (16)	C8—C9	1.3930 (19)
N2—H3n	0.888 (9)	C9—C10	1.3902 (18)
C1—C5	1.5425 (18)	C9—H9	0.9500
C1—C2	1.5771 (16)	C10—C11	1.394 (2)
C1—H1	1.0000	C10—H10	0.9500
C2—C3	1.5502 (18)	C11—C12	1.3977 (19)
C2—H2	1.0000	C11—C14	1.5076 (18)
C3—C7	1.5347 (19)	C12—C13	1.3925 (19)
C3—C4	1.5376 (19)	C12—H12	0.9500
C3—H3	1.0000	C13—H13	0.9500
C4—C5	1.5432 (18)	C14—H14A	0.9800
C4—H4A	0.9900	C14—H14B	0.9800
C4—H4B	0.9900	C14—H14C	0.9800
C5—C6	1.537 (2)		
			
O2—S1—O1	119.52 (6)	C6—C5—H5	114.4
O2—S1—N2	105.89 (6)	C1—C5—H5	114.4
O1—S1—N2	108.41 (6)	C4—C5—H5	114.4
O2—S1—C8	108.84 (6)	C5—C6—C7	103.51 (11)
O1—S1—C8	105.56 (6)	C5—C6—H6A	111.1
N2—S1—C8	108.23 (6)	C7—C6—H6A	111.1
C1—N1—H1n	112.5 (12)	C5—C6—H6B	111.1
C1—N1—H2n	111.1 (13)	C7—C6—H6B	111.1
H1n—N1—H2n	100.2 (18)	H6A—C6—H6B	109.0
C2—N2—S1	120.32 (9)	C3—C7—C6	102.75 (11)
C2—N2—H3n	120.9 (14)	C3—C7—H7A	111.2
S1—N2—H3n	110.2 (14)	C6—C7—H7A	111.2
N1—C1—C5	117.79 (10)	C3—C7—H7B	111.2
N1—C1—C2	114.02 (10)	C6—C7—H7B	111.2
C5—C1—C2	102.34 (10)	H7A—C7—H7B	109.1
N1—C1—H1	107.4	C13—C8—C9	120.92 (13)
C5—C1—H1	107.4	C13—C8—S1	120.39 (10)
C2—C1—H1	107.4	C9—C8—S1	118.69 (11)
N2—C2—C3	115.36 (10)	C10—C9—C8	119.41 (13)
N2—C2—C1	112.93 (10)	C10—C9—H9	120.3
C3—C2—C1	102.95 (10)	C8—C9—H9	120.3
N2—C2—H2	108.4	C9—C10—C11	120.88 (13)
C3—C2—H2	108.4	C9—C10—H10	119.6
C1—C2—H2	108.4	C11—C10—H10	119.6
C7—C3—C4	101.26 (11)	C10—C11—C12	118.56 (12)
C7—C3—C2	109.72 (11)	C10—C11—C14	120.16 (13)
C4—C3—C2	101.29 (10)	C12—C11—C14	121.25 (13)
C7—C3—H3	114.4	C13—C12—C11	121.36 (13)
C4—C3—H3	114.4	C13—C12—H12	119.3
C2—C3—H3	114.4	C11—C12—H12	119.3
C3—C4—C5	94.26 (10)	C8—C13—C12	118.85 (12)
C3—C4—H4A	112.9	C8—C13—H13	120.6
C5—C4—H4A	112.9	C12—C13—H13	120.6
C3—C4—H4B	112.9	C11—C14—H14A	109.5
C5—C4—H4B	112.9	C11—C14—H14B	109.5
H4A—C4—H4B	110.3	H14A—C14—H14B	109.5
C6—C5—C1	109.75 (11)	C11—C14—H14C	109.5
C6—C5—C4	102.60 (11)	H14A—C14—H14C	109.5
C1—C5—C4	99.82 (10)	H14B—C14—H14C	109.5
			
O2—S1—N2—C2	−174.49 (10)	C1—C5—C6—C7	−74.78 (12)
O1—S1—N2—C2	56.10 (11)	C4—C5—C6—C7	30.64 (13)
C8—S1—N2—C2	−57.93 (11)	C4—C3—C7—C6	−39.25 (12)
S1—N2—C2—C3	93.87 (12)	C2—C3—C7—C6	67.22 (13)
S1—N2—C2—C1	−148.11 (9)	C5—C6—C7—C3	5.12 (13)
N1—C1—C2—N2	8.26 (15)	O2—S1—C8—C13	32.18 (13)
C5—C1—C2—N2	−120.07 (12)	O1—S1—C8—C13	161.63 (11)
N1—C1—C2—C3	133.32 (11)	N2—S1—C8—C13	−82.46 (12)
C5—C1—C2—C3	5.00 (12)	O2—S1—C8—C9	−147.16 (10)
N2—C2—C3—C7	49.21 (14)	O1—S1—C8—C9	−17.71 (12)
C1—C2—C3—C7	−74.26 (12)	N2—S1—C8—C9	98.19 (11)
N2—C2—C3—C4	155.65 (10)	C13—C8—C9—C10	−0.4 (2)
C1—C2—C3—C4	32.18 (12)	S1—C8—C9—C10	178.91 (10)
C7—C3—C4—C5	56.71 (12)	C8—C9—C10—C11	0.0 (2)
C2—C3—C4—C5	−56.28 (12)	C9—C10—C11—C12	0.5 (2)
N1—C1—C5—C6	−58.83 (14)	C9—C10—C11—C14	−177.73 (12)
C2—C1—C5—C6	67.07 (12)	C10—C11—C12—C13	−0.68 (19)
N1—C1—C5—C4	−166.13 (11)	C14—C11—C12—C13	177.57 (12)
C2—C1—C5—C4	−40.23 (12)	C9—C8—C13—C12	0.3 (2)
C3—C4—C5—C6	−53.44 (12)	S1—C8—C13—C12	−179.03 (10)
C3—C4—C5—C1	59.52 (12)	C11—C12—C13—C8	0.3 (2)

### Hydrogen-bond geometry (Å, º)

**Table:**

*D*—H···*A*	*D*—H	H···*A*	*D*···*A*	*D*—H···*A*
N1—H1*n*···O1^i^	0.88 (1)	2.20 (1)	2.976 (2)	148 (2)
N1—H2*n*···N2	0.87 (1)	2.39 (2)	2.752 (2)	105 (2)
N2—H3*n*···N1^i^	0.89 (1)	2.04 (1)	2.907 (2)	166 (2)

Symmetry code: (i) −*x*+1, *y*−1/2, −*z*+2.
